# Skin Lightening Practices and Associated Factors Among Undergraduate Female University Students in Kilimanjaro Region, Northern Tanzania: A Cross‐Sectional Study

**DOI:** 10.1111/jocd.71293

**Published:** 2026-11-13

**Authors:** Issaya Kweka, John Mtenga, Mariam Chumo, Jackline Njau, Lameck Pashet Sengeruan, Daniel E. Mwakasaka, Sia Saria, Jere Mshana, Rachel Manongi

**Affiliations:** ^1^ KCMC University Moshi Tanzania; ^2^ Kishapu District Council Shinyanga Tanzania; ^3^ Dermatology Department KCMC University Moshi Tanzania; ^4^ School of Public Health KCMC University Moshi Tanzania

**Keywords:** cross‐sectional study, prevalence, skin lightening practices, skin lightening products, Tanzania, undergraduate university students

## Abstract

**Background:**

Unofficial use of skin lightening products (SLPs) for cosmetic purposes is a rising public health concern. In Tanzania, the challenges in regulating SLP retail outlets have allowed products containing harmful ingredients to remain accessible, posing particular risks to young women, including university students.

**Study Aim:**

To determine the prevalence of skin lightening practices and associated factors among undergraduate female university students in the Kilimanjaro region, northern Tanzania.

**Methodology:**

A cross‐sectional study was conducted from May 2024 to June 2024 among undergraduate female students from three universities in Moshi Urban District, Kilimanjaro region. Multistage stratified sampling, proportionate to size, was used to select 414 participants. Data were collected using an online, self‐administered, structured questionnaire via Kobo toolbox. Chi‐square test was used to determine significant differences in proportions across categories of the variables, and perceived colorism among SLP users and non‐users was analyzed using two‐tailed *t*‐tests. Furthermore, multiple logistic regression was applied to determine factors associated with the use of SLPs.

**Results:**

The prevalence of SLPs use among participants was 45.5% (95% CI: 40.8%–50.4%). Creams were the most commonly used form (52.1%), followed by the combined use of soaps and gels (23.4%). Composite colorism scores were significantly higher in SLP users than non‐users (17.54 vs. 13.87; *p* < 0.001). Factors independently associated with SLPs use were fair skin color (AOR = 2.1; 95% CI: 1.33–3.43; *p* = 0.002), white skin color (AOR = 1.99; 95% CI: 1.06–3.71; *p* = 0.031), and off‐campus residence (AOR = 1.7; 95% CI: 1.12–2.69; *p* = 0.014).

**Conclusion:**

Nearly half of the undergraduate female students surveyed used SLPs, with a significant proportion using banned products containing harmful ingredients such as hydroquinone and corticosteroids, often in combination. These findings highlight the crucial need for stronger regulatory enforcement by the Tanzania Bureau of Standards, and for the studied universities to implement targeted educational programmes that promote skin health and behavior change among female students in order to promote evidence‐based, safer beauty choices among students.

## 1 Background

Skin lightening for cosmetic purposes refers to the use of natural or synthetic substances to lighten skin tone or achieve an even complexion [1, 2]. Long‐term use of SLPs containing harmful ingredients such as hydroquinone, mercury, corticosteroids, or high concentrations of kojic acid reduces melanin concentration in the skin and leads to physiological pigment reduction. Moreover, the use of such products is associated with steroid‐induced acne, striae, telangiectasia, cutaneous atrophy, hirsutism, and opportunistic bacterial, fungal, and viral infections [3, 4, 5]. Systemic complications including hypertension, nephrotoxicity, neurotoxicity, diabetes, hormonal disruption (for instance, altered estrogen levels), and an increased risk of skin cancer have also been documented, with significant negative impacts on quality of life [3, 6].

The skin cosmetics industry is growing rapidly. Although both men and women use SLPs, women do so at substantially higher rates, particularly in Africa, Asia, the Middle East, and America, driven by personal, social, and cultural preferences [7, 8, 9]. The global market for SLPs is estimated at USD 8.8 billion per year, with projections suggesting it will reach USD 15.7 billion by 2030 [10, 11]. The pooled prevalence of ever‐use of SLPs across studied populations is approximately 27.7% [12], with Africa reporting the highest regional prevalence at 53.6% [9].

Multiple studies have reported SLPs use among women across diverse educational levels, with different prevalences being reported [13, 14, 15]. Several SLP ingredients, including mercury, hydroquinone, and corticosteroids, have been banned in multiple countries, including Tanzania, owing to their adverse health effects [16]. In the United Kingdom, for example, two women sustained kidney damage attributable to the use of mercury‐containing skin creams [17].

In Tanzania, the use of SLPs among women for beautification purposes has been increasing [18], while regulatory agencies continue to face challenges in enforcing restrictions on the sale of harmful products across different retail channels.

University students represent a particularly important subgroup for investigation. Students in higher learning institutions report cosmetics use at higher rates (51.1%) than the general population (36.8%) globally [9], a pattern attributed to heightened media exposure, peer influence, and the social pressures associated with transitioning into adulthood in a new environment. The university period is also characterized by increased independence and opportunities for social interaction, factors that may facilitate the adoption of cosmetic practices including skin lightening. In Tanzania, a study done in Dar es Salaam reported that 40% of 112 health science students used SLPs for beautification [19]. However, SLPs use among university students specifically has not been examined in the Kilimanjaro region, and no study has focused exclusively on this subgroup across multiple disciplines and institutions.

Local studies on skin lightening practices across different subgroups are essential because the practice is shaped by cultural, economic, and historical factors that vary by context, and such data are critical for guiding targeted interventions and regulatory enforcement. Therefore, this study aimed to determine the prevalence of skin lightening practices and the factors associated with SLPs use among undergraduate female university students in the Kilimanjaro region, northern Tanzania.

## 2 Materials and Methods

### 2.1 Study Design, Area and Participants

This was a cross‐sectional study conducted in Moshi Urban District, Kilimanjaro region, northern Tanzania, from May to June 2024. Kilimanjaro region shares borders with Arusha region, Tanga region, and Kenya, and comprises seven districts: Same, Hai, Rombo, Siha, Mwanga, Moshi Urban, and Moshi Rural. At the time of the study, Moshi Urban District hosted four universities: Moshi Cooperative University (MoCU), Mwenge Catholic University (MWECAU), KCMC University, and Stefano Moshi Memorial University College (SMMUCo) [20].

MoCU is a public university offering undergraduate programmes, including Accounting and Finance Co‐operative Management, Business Economics, Procurement and Supply Management, Human Resource Management, Information and Communication Technology, and Law. MWECAU is a private institution offering programmes, including Education, Business Administration, Environmental Studies, Sociology and Social Work, Mathematics and Statistics, Philosophy, Project Planning and Management, Computer Science, Chemistry, Applied Biology, and Law. KCMC University is a private institution offering health‐related undergraduate programmes, including Doctor of Medicine, Nursing, Prosthetics and Orthotics, Physiotherapy, Health Laboratory Sciences, Optometry, and Occupational Therapy. SMMUCo is a private college affiliated with Tumaini University of Makumira, offering Community Development, Business Administration, and Education programmes.

### 2.2 Sample Size, Sampling Strategy and Participants Selection

#### 2.2.1 Sample Size Calculation

The target minimum sample size was calculated using the standard Cochran formula:

$$n=z^{2}\times p\times q/e^{2}$$

where *p* = 0.409, prevalence estimate from published literature [21]; *q* = 0.591, *q* = 1 – *p*; *z* = 1.96 (95% confidence level); *e* = 0.05 (5% margin of error). This yielded a minimum sample size of *n* = 372 participants. Adjusting for an anticipated 10% non‐response rate, the final required sample size was 414.

#### 2.2.2 Sampling Strategy

Of the four universities in Moshi Urban District, three were eligible for inclusion based on offering comparable non‐medical undergraduate programmes: MoCU, MWECAU, and SMMUCo. Simple random sampling was utilized to select two of the three universities (lottery method): MoCU and MWECAU. KCMC University, as the only medical university in the area, was purposively added to ensure disciplinary diversity and a clinically trained comparison group. SMMUCo was therefore not included, having not been selected in the random draw. The study, thus, enrolled participants from MoCU, MWECAU, and KCMC University.

#### 2.2.3 Participants Selection

Multistage stratified sampling proportionate to size was applied across three stages. In Stage 1, participants were stratified by university. In Stage 2, they were further stratified by course of study. In Stage 3, participants were stratified by the year of study, from which they were randomly selected. The allocation formula used was:

$$\mathrm{nᵢ}=\left(\mathrm{Nᵢ}/N\right)\times n$$

where *nᵢ* = sample size for a stratum *i*; *Nᵢ* = total number of female students in a stratum *i*; *N* = total number of female students in the respective university; and *n* = overall sample size 414.

The total number of eligible female undergraduate students across the three study sites was 5936 (KCMC University: 820; MWECAU: 2478; MoCU: 2638). Therefore, 58 participants were selected from KCMC University, 172 from MWECAU, and 184 from MoCU. At each institution, courses were selected purposively to get a representation of diverse disciplines: four courses at KCMC University (Doctor of Medicine, Nursing, Health Laboratory Sciences, and Physiotherapy), three at MWECAU (Arts with Education, Science with Education, and Science in Chemistry), and three at MoCU (Finance and Accounting, Human Resource Management, and Law). Participants who self‐reported having a history of skin medical conditions such as skin allergies and skin cancer were excluded from the study. SLPs use was defined as self‐reported current or recent use of any skin‐lightening product within the 12 months prior to data collection.

The study sample comprised undergraduate female students who were registered at the time of data collection and provided written informed consent. Male students were not included, as the study was specifically designed to examine practices among female students given the disproportionately higher prevalence of SLPs use in this group. Figure 1 illustrates the participant selection process by year of study across courses and universities.

**FIGURE 1:**
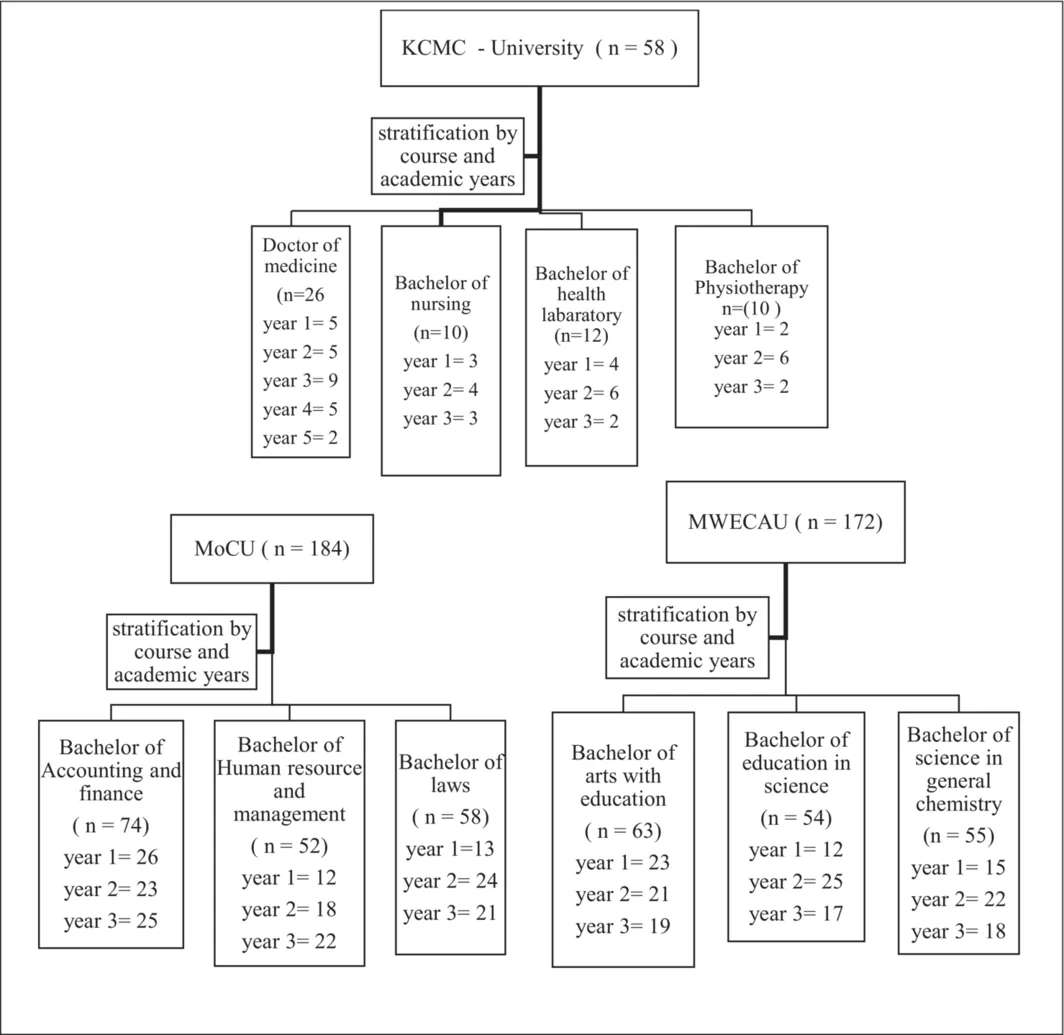
Showing selection of participants per year of study across courses of study.

#### 2.2.4 Data Collection Instrument and Procedure

A structured, self‐administered questionnaire was developed in English, the medium of instruction in Tanzanian higher learning institutions. The questionnaire covered three domains: (i) socio‐demographic characteristics; (ii) use of skin lightening products; and (iii) behavioral practices associated with SLP use. Attitudinal perceptions (colorism statements) on SLPs use such as self‐esteem, beauty, marriageability, high social class and younger appearance were assessed by asking participants to score 5 statements on a Likert scale of 1 (strongly disagree) to 5 (strongly agree). The scores were summed into a composite colorism score ranging from 5 to 25, with higher scores signifying stronger perceived colorism.

Prior to data collection, a pilot study was conducted among female students from two non‐enrolled institutions (Kilimanjaro College of Health and Allied Sciences and Kilimanjaro School of Pharmacy) to assess clarity and face validity. In addition, a brief survey of cosmetic shops near the study campuses was conducted to identify locally available SLPs, and this information was used to supplement the response options in the questionnaire. The finalized questionnaire was administered electronically via Kobo Toolbox and distributed to the selected participants through their respective university contacts.

#### 2.2.5 Data Management and Statistical Analysis

Collected data were coded, entered into Microsoft Excel, and exported to Stata version 14 (StataCorp LLC, College Station, TX, USA) for analysis. Descriptive statistics were used to characterize the study sample and summarize the prevalence and patterns of SLPs use. Chi‐square test was applied to assess statistically significant differences in SLPs use across universities and among participants according to skin color, and a two‐tailed *t*‐test was used to compare perceived colorism among SLP users and non‐users.

For multivariable analysis, each explanatory variable was first assessed in univariate logistic regression. Variables with a *p*‐value ≤ 0.2 in univariate analysis were candidates for inclusion in the multivariable logistic regression model. Variables considered a priori to be clinically or contextually relevant were also retained regardless of *p*‐value. Regression results are presented as crude odds ratios (CORs) and adjusted odds ratios (AORs) with 95% confidence intervals (CIs). Statistical significance in multivariable analysis was defined as a two‐tailed *p*‐value < 0.05.

### 2.3 Ethical Approval

The KCMC University Ethics Committee approved the study (UG27/2024). After that, each participating university received the proposal to obtain a study permit. Participation was voluntary, and each participant provided informed consent. Participants were also informed that they were free to discontinue their involvement in the study at any time.

## 3 Results

### 3.1 Socio‐Demographic Attributes of Participants

Of the 414 sampled participants, 413 provided complete responses, yielding a response rate of 99.8%. The median age of participants was 21.9 years (IQR: 20.7–23.7). Majority (355/413; 85.9%) were enrolled in non‐health‐related courses. The overall prevalence of SLPs use was 45.5% (95% CI: 40.8%–50.4%; 188/413). Prevalence by university is shown in Figure 2.

**FIGURE 2:**
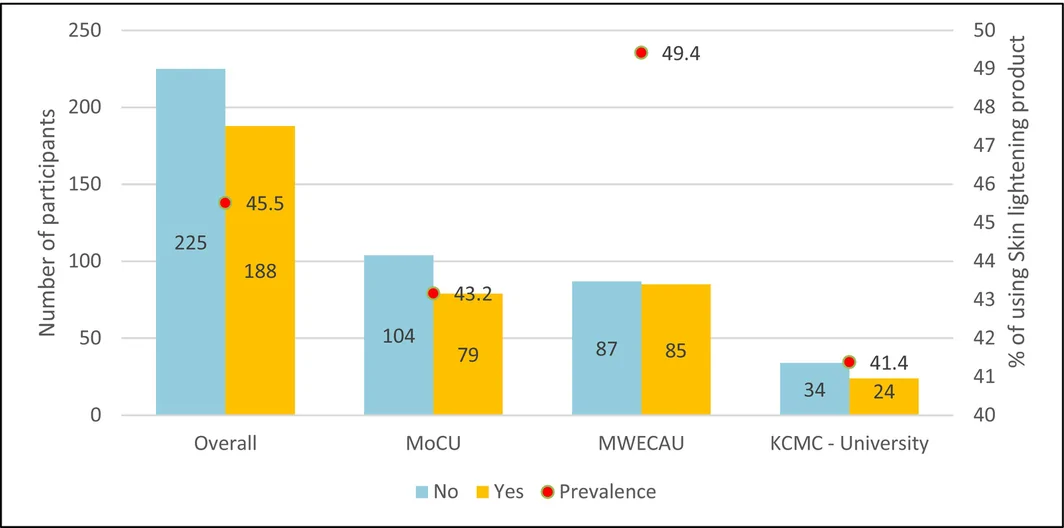
Proportion of SLPs usage among study participants in overall and per university.

More than half of participants (221/413; 53.5%) had a fair skin tone, 62.7% (259/413) lived off‐campus, and the largest proportion (269/413; 65.1%) were junior students. Socio‐demographic characteristics are summarized in Table 1.

**TABLE 1:** Socio‐demographic characteristics of the study participants (*n* = 413).

Variable	Frequency (*n*)	Percentage (%)
Median age in years (IQR): 21.9 (20.7–23.7)		
Skin color		
Fair	221	53.5
Dark	124	30.0
White	68	16.5
University		
KCMC	58	14.1
MoCU	183	44.3
MWECAU	172	41.6
Categorized year of study		
Junior students	269	65.1
Senior students	144	34.9
Marital status		
Married	19	4.6
Single	394	95.4
Grew up area		
Rural	133	32.2
Urban	280	67.8
Residence area during studies		
On‐campus	154	37.3
Off‐campus	259	62.7
Participants' religion		
Christians	321	77.8
Muslim	91	22.0
Hindu	01	0.2
Type of courses being admitted		
Health and allied science	58	14.0
Non‐health science	355	86.0
Cosmetics usage status		
No	225	54.5
Yes	188	45.5

*Note:* Junior students = Year 1 and Year 2 students; Senior = Year 3, Year 4 and Year 5.

### 3.2 Behavioral Practices Related to Skin Lightening Products Usage Among SLP Users (*n* = 188)

Among the 188 SLP users, the most commonly used product form was creams (98/188; 52.1%), followed by the combined use of soaps and gels (44/188; 23.4%). More than half of participants (108/188; 57.5%) reported using SLPs five or more times in the preceding 12 months, and 80.3% (151/188) applied them daily. Awareness of product ingredients was low: 84.6% were unaware of the active ingredients in the products they used, and 74.5% did not read product labels before purchase. Participants obtained information about SLPs from multiple simultaneous sources, most commonly friends or acquaintances and social media or television. The majority (87.8%) purchased products from cosmetic shops. Detailed behavioral data are presented in Table 2.

**TABLE 2:** Behavioral practices related to SLPs use among participants (*n* = 188).

Variable	Frequency (*n*)	Percentage (%)
Types of skin lightening products used		
Creams	98	52.1
Creams, soaps and gel	33	17.6
Soap and gel	44	23.4
Other combinations^a^	13	6.9
Number of times SLPs used in the last 12 months		
Once	45	23.9
Twice	15	7.9
Thrice	11	5.9
Four times	09	4.8
Five times or more	108	57.5
Frequency of skin lightening product use		
Everyday	151	80.3
Every week	14	7.4
Every month	07	3.7
Every 3 months	10	5.3
More than every 3 months	06	3.3
Reading of ingredient labels before purchasing		
No	140	74.5
Yes	48	25.5
Aware of active ingredients in products used		
No	159	84.6
Yes	29	15.4
Primary source of information about SLPs^b^		
Friends/acquaintance/media/television/radio	54	28.7
Media/television/radio only	49	26.1
Friends/acquaintance only	35	18.6
Family/social media/television/radio	11	5.9
Other sources^c^	39	20.7
Perceived affordability of skin lightening products		
Affordable	92	48.9
Expensive	96	51.1
Place of purchase		
Cosmetic shops	165	87.8
Drug store/pharmacy	05	2.7
Hospitals/clinics	04	2.0
Online	13	7.0
Traditional medical practitioner	01	0.5

^a^ Other combinations: soap, gel and capsules; creams and capsules.

^b^ Participants could indicate multiple simultaneous sources.

^c^ Other sources include: family members alone; family members and acquaintances; healthcare personnel; books/literature.

### 3.3 Skin Lightening Agents Used by Participants

More than a quarter of SLP users (51/188; 27.1%) used multiple agents in combination. Combination regimens included Turmeric with Kojic acid; Turmeric with Golden Glow; Turmeric, Kojic acid, and Fair & White Cream; Palmer's Skin Success Cream, Kojic acid, and Turmeric; Perlay Goldie and Turmeric; Palmer's Skin Success Cream and Extra Clear Cream; Golden Glow, Turmeric, and Soft & Beautiful Cream; and Bronz Tone, Golden Glow, Kojic acid, Turmeric, Palmer's Skin Success Cream, Fair & White Cream, and Perlay Goldie. Notably, many of these agents are banned in Tanzania and in several European countries owing to safety concerns. Other products used by participants included Vitamin C Serum, Vitamin E, Cocoa Butter, Garnier, Epiderm, Pearl Essence, Snail Cream, White Gold, Argan Oil, and Vaseline, among others. The full distribution of named products is shown in Figure 3.

**FIGURE 3:**
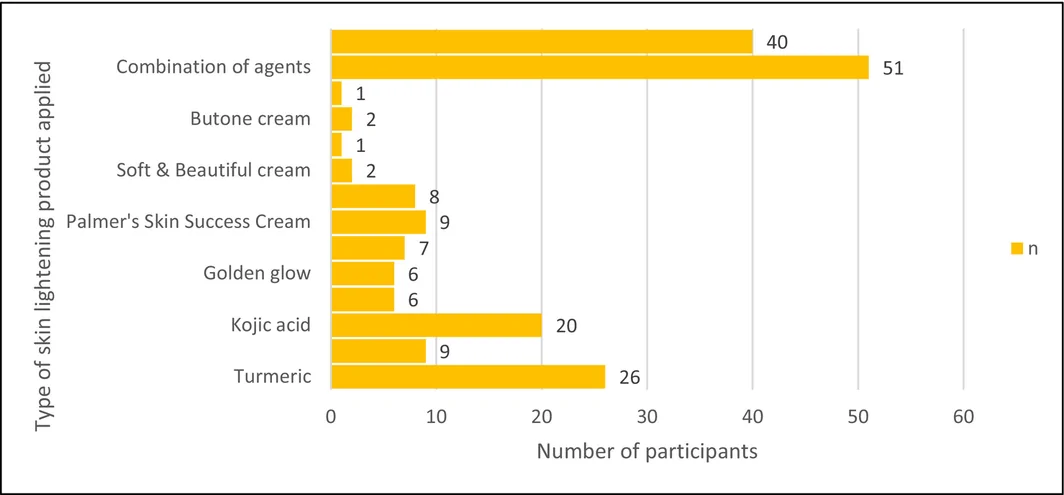
Skin lightening products by names reported by participants.

### 3.4 Factors Considered During Product Purchase, Perceptions and Consequence Awareness

Table 3 shows the factors considered by SLP users when purchasing products and their perceptions about skin lightening. The most common purchasing consideration was a combination of product brand name and perceived rapid effectiveness (52/188; 27.7%), followed by brand name alone (38/188; 20.2%) and a combination of brand name, reasonable price, and a friend's recommendation (34/188; 18.1%). Very few participants (13/188; 6.9%) considered product ingredients when making purchase decisions.

**TABLE 3:** Factors considered during purchase of SLPs and perceptions among SLP users (*n* = 188).

Variable	Frequency (*n*)	Percentage (%)
Factors considered when purchasing SLPs		
Product advertisement only	3	1.6
Friend's recommendation only	6	3.1
Reasonable price only	11	5.9
Ingredients of the product	13	6.9
Brand name only	38	20.2
Brand and rapid effectiveness	52	27.7
Brand, price and friend's recommendation	34	18.1
Rapid effectiveness and media advertisement	31	16.5
Perceptions about skin lightening (*n* = 188 SLP users)		
Lighter skin tone provides higher self‐esteem		
Strongly agree	63	33.5
Agree	85	45.2
Neutral	21	11.2
Disagree	14	7.4
Strongly disagree	5	2.7
Lighter skin tone gives a younger appearance		
Strongly agree	53	28.2
Agree	82	43.7
Neutral	32	17.0
Disagree	17	9.0
Strongly disagree	4	2.1
Lighter skin tone implies belonging to a high social class		
Strongly agree	29	15.4
Agree	44	23.4
Neutral	30	16.0
Disagree	46	24.5
Strongly disagree	39	20.7
Lighter skin tone increases chances of marriage		
Strongly agree	22	11.7
Agree	32	17.0
Neutral	41	21.8
Disagree	41	21.8
Strongly disagree	52	27.7
People regard a lighter skin tone as more beautiful		
Strongly agree	52	27.7
Agree	92	49.0
Neutral	23	12.2
Disagree	14	7.4
Strongly disagree	7	3.7

Among SLP users, strong agreement or agreement was prevalent across three key perceptual domains: increased self‐esteem (78.7%), implications of beauty (76.6%), and a more youthful appearance (71.9%). Fewer participants endorsed social‐class (38.8% agree/strongly agree) or marriageability (28.7% agree/strongly agree) dimensions of skin color. Participants' awareness of the consequences of SLP use is summarized in Figure 4.

**FIGURE 4:**
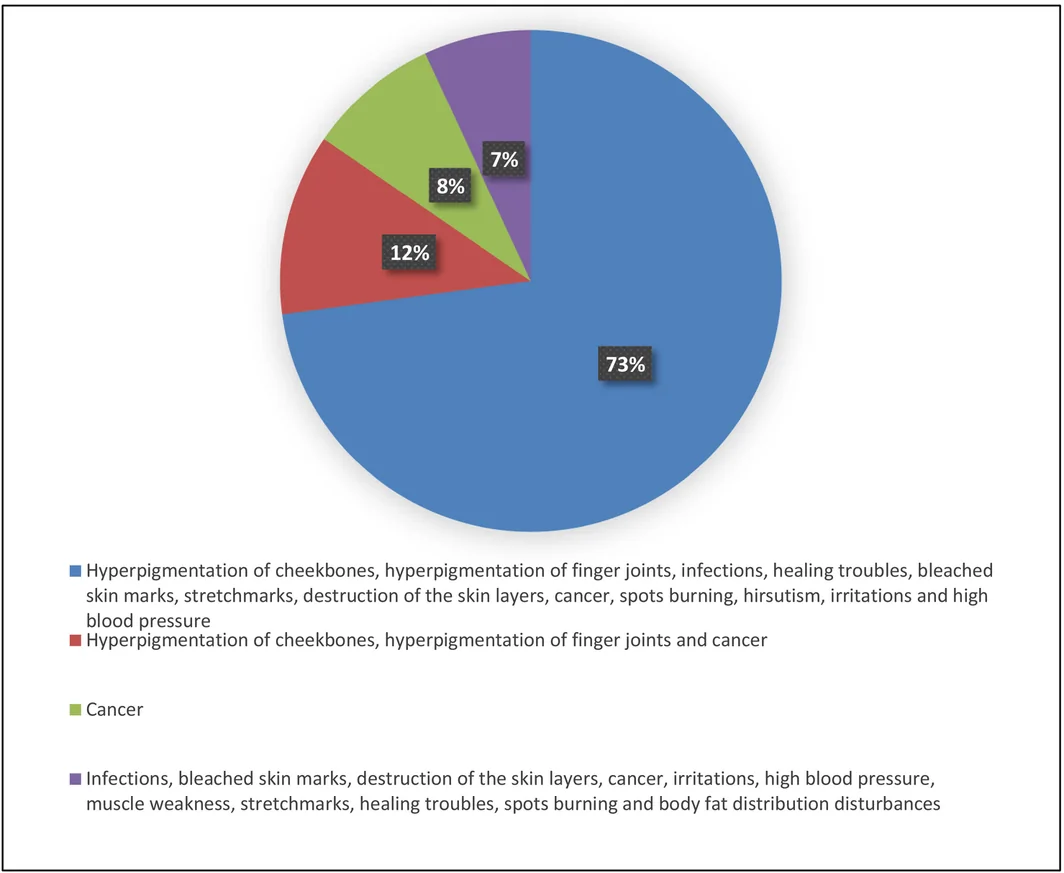
Participants' awareness on the consequences of skin lightening product use.

### 3.5 Comparison of Colorism Scores Among Skin Lightening Users and Non‐Users

Composite colorism scores were significantly higher in those that used SL agents than in those that did not (17.54 vs. 13.87; *p* < 0.001). Skin lightening users more strongly agreed with all statements that lighter skin provides higher self‐esteem (*p* < 0.001), gives a younger appearance (*p* < 0.001), implies belonging to a high social class (*p* < 0.001), increases one's chance of getting married (*p* < 0.001), and that the lighter skin is more beautiful (*p* < 0.001) (Table 4).

**TABLE 4:** Comparison of colorism scores among skin lightening users (*n* = 188) and non‐users (*n* = 225).

Colorism statements	Average score	Average score	*p*
	Skin lightening users	Non skin lightening users	
Lighter skin tone provides higher self‐esteem	3.99	3.14	< 0.001
Lighter skin tone gives a younger appearance	3.87	3.01	< 0.001
Lighter skin tone implies belonging to a high social class	2.88	2.09	< 0.001
Lighter skin tone increases chances of marriage	2.63	1.97	< 0.001
People regard a lighter skin tone as more beautiful	4.17	3.66	< 0.001
Composite score	17.54	13.87	< 0.001

### 3.6 Distribution of SLPs Use Across Participant Subgroups

There were no statistically significant differences in SLPs use across universities *χ* ^2^ (2, *n* = 413) = (1.86, *p* = 0.394) or between health‐related and non‐health‐related course enrolments *χ* ^2^ (1, *n* = 413) = (0.467, *p* = 0.495). By contrast, significant differences in SLPs use were observed according to skin color *χ* ^2^ (2, *n* = 413) = (8.40, *p* = 0.015), marital status *χ* ^2^ (1, *n* = 413) = (4.21, *p* = 0.04), and area of residence during university studies *χ* ^2^ (1, *n* = 413) = (9.52, *p* = 0.002).

### 3.7 Determinants of Skin‐Lightening Products (SLPs) Use Within the Study Population

Table 5 presents crude and adjusted odds ratios for factors associated with SLPs use. In multivariable analysis, fair skin color was independently associated with 2.2‐fold higher odds of SLPs use compared with dark skin (AOR = 2.1; 95% CI: 1.33–3.43; *p* = 0.002), and white skin color with 1.99‐fold higher odds (AOR = 1.99; 95% CI: 1.06–3.71; *p* = 0.031). Off‐campus residence was associated with 60% higher odds of SLPs use compared with on‐campus residence (AOR = 1.7; 95% CI: 1.12–2.69; *p* = 0.014). Years of study, marital status, area of upbringing, and type of course enrolled were not independently associated with SLP use in multivariable analysis.

**TABLE 5:** Factors associated with SLPs use among participants (*n* = 413).

Univariate analysis				Multivariable analysis		
Variable	CO*R* ^a^	95% CI	*p*	AO*R* ^b^	95% CI	*p*
Marital status						
Single (Ref.)	__	__	__	__	__	__
Married	2.7	1.01–7.3	0.048	2.2	0.77–6.25	0.14
Skin color						
Dark skin (Ref.)	__	__	__	__	__	__
Fair skin	1.90	1.21–2.99	0.006	2.1	1.33–3.43	0.002
White skin	1.89	1.03–3.44	0.039	1.99	1.06–3.71	0.031
Area of residence						
On‐campus (Ref.)	__	__	__	__	__	__
Off‐campus	1.9	1.26–2.86	0.002	1.7	1.12–2.69	0.014
Years of study						
Senior students	__	__	__	__	__	__
Junior students	0.7	0.44–1.00	0.051	0.8	0.53–1.28	0.39
Area where participant grew up						
Urban (Ref.)	__	__	__	__	__	__
Rural	0.7	0.45–1.03	0.071	0.77	0.49–1.19	0.25
Type of course enrolled						
Health related (Ref.)	__	__	__	__	__	__
Non‐health related	1.22	0.69–2.14	0.495	1.28	0.72–2.29	0.40

Abbreviations: Ref., reference category; CI, confidence interval.

^a^ Crude odds ratio.

^b^ Adjusted odds ratio.

## 4 Discussion

The present study establishes the prevalence of skin‐lightening products (SLPs) use of 45.5% among undergraduate female university students in Kilimanjaro region, Tanzania. Although the dermatological application of SLPs is clinically indicated for hyperpigmentation disorders (e.g., melasma) when supervised by medical professionals, the current study focused exclusively on cosmetic use among students without known skin medical conditions. Studies on this specific subgroup have been limited in Tanzania, and the present findings contribute important local evidence to guide interventions and regulatory responses.

The prevalence of 45.5% observed in this study is substantially lower than the 91% reported in a previous Tanzanian study of undergraduate health science students in Dar es Salaam [19]. The difference in prevalences may reflect the distinct study settings, populations, and data collection periods. Notably, the Dar es Salaam study enrolled both male and female students from a single faculty, whereas the present study enrolled female students from diverse disciplines across three universities. Despite this difference, the prevalence in our study is broadly comparable to findings from Nigeria (40.9%) [22], Ghana (40.9%) [21] and Jordan (40.7%) [14], supporting the argument that SLPs use among university students is a prevalent, cross‐cultural phenomenon driven by shared social and aesthetic norms.

Creams were the most frequently used SLPs form (52.1%), consistent with reports from Ghana [21] and Jordan [14], where creams were also the predominant form. Community‐based studies in Tanzania and elsewhere have similarly reported high cream use [23, 24]. The preference for creams may reflect practical advantages including ease of application to the targeted body areas, perceived high efficacy, the higher concentration of active ingredients in cream formulations, and their role in protecting the skin barrier during active ingredient absorption [25].

A striking finding is that 80.3% of SLP users applied products daily, a proportion which is considerably higher than the 55.6% reported among medical students in Jordan [14]. The high frequency and intensity use of the SLPs documented in this study with 57.5% of users reported use five or more times in the past 12 months, and 80.3% applied products every day carries significant public health implications. Prolonged, high‐frequency exposure to SLPs containing harmful ingredients substantially increases the risk of both local dermatological complications and systemic toxicity [3, 6]. The findings revealed significant deficits in consumer awareness, with 84.6% of users reporting ignorance regarding their products' active ingredients, and 74.5% purchasing products without prior label inspection; this compounds this risk and highlights a critical knowledge gap that educational interventions should address.

More than a quarter of SLP users (27.1%) applied agents in combination, a finding which is consistent with a report from Nigeria [4], where combination use was motivated by the expectation of enhanced efficacy. However, combining SLPs, particularly those containing prohibited concentrations of active ingredients, increases the risk of toxicity and chronic skin inflammation [8, 26]. Several banned products identified in the present study, including Fair & White Cream, Palmer's Skin Success Cream, Extra Clear Cream, and Soft & Beautiful Cream, all of which are prohibited in Tanzania for containing hydroquinone or corticosteroids [16], were actively being used by participants. Additionally, Perlay Goldie, which is banned in the European Union owing to health‐related risks [27, 28], was used by a subset of participants. These findings point to the likely existence of an informal black market for banned products, weaknesses in regulatory enforcement, the presence of online sales channels that circumvent formal oversight, and possible product mislabelling or rebranding.

Participants obtained information about SLPs from a range of sources, most commonly from friends, acquaintances, and social media or broadcast media, often simultaneously [11]. Reliance on multiple informal and unregulated sources may result in fragmented or inaccurate information about the health risks of SLPs [29, 30], reinforcing the need for evidence‐based public health messaging through trusted channels. All SLPs‐related advertising in Tanzania should be monitored and regulated to ensure that only products meeting safety standards are promoted.

A counter‐intuitive but noteworthy finding is that participants with fair or white skin tone had significantly higher odds of SLP use than those with dark skin. A comparable finding was reported among female medical undergraduates in Nigeria, where lighter‐skinned participants were more likely to use SLPs than darker‐skinned peers [22]. This pattern may reflect a motivational difference: whereas darker‐skinned individuals may use SLPs to achieve a lighter complexion, fair‐ and white‐skinned individuals may use them primarily to maintain their current skin tone, or to address uneven pigmentation, rather than to achieve a dramatic color change. Additionally, colorism—the social preference for lighter skin tones linked historically to higher social status, employability, perceived beauty, and marriageability [11, 31, 32], may create social pressure on fair‐skinned individuals to preserve or enhance their complexion. The high levels of agreement in this study that lighter skin provides self‐esteem (78.7%) and implies beauty (76.6%) support this interpretation.

The finding that course type (health‐related versus non‐health‐related) was not independently associated with SLPs use is noteworthy, as health science students might be expected to have greater awareness of the risks of SLP use; however, awareness alone may not be sufficient to change cosmetic behavior.

The proportion of participants who were aware of the ingredients present in skin lightening products was small (15.4%). In addition, 92% of the participants mentioned multiple adverse effects related to the application of skin lightening products, and 74.5% of them did not read on the ingredients of the products before making a decision to purchase. This finding corroborates with reports by Yesaya et al. and Tesfamariam et al. that showed 83.9% and 80.1% of the users don't read on the ingredients of SLPs, respectively [23, 24], supporting a statement that skin lightening practices are influenced by personal, peer pressure, social and cultural factors [8, 11, 23], regardless of the consequences. Therefore, planned educational programmes on raising awareness about reading products' ingredients among students are necessary for them to make informed health choices.

Users of skin lightening products had a significantly stronger belief in colorism than nonusers, that lighter skin increased self‐esteem, gives younger appearance, implies belonging to high social class, increased chances of marriageability, increased romantic prospects and beautifulness (*p* < 0.001). These findings are consistent with similar studies conducted previously among women in Tanzania [23] and Jordan [14], who engaged in the use of SLPs.

Living off‐campus was associated with 70% higher odds of SLPs use compared with on‐campus residence. Off‐campus students may have greater autonomy, reduced institutional oversight, and more opportunities for social interaction with the wider community, all of which may facilitate the adoption and maintenance of cosmetic practices driven by appearance norms [33]. Additionally, off‐campus students have easier physical access to cosmetic shops, where the majority of SLPs (87.8%) were purchased in this study.

## 5 Study Limitations

The study used a cross‐sectional study design which precludes causal inference; the associations identified between the explanatory variables and SLPs use do not establish temporal or causal relationships. Also, data collection depended on self‐reporting which may have introduced potential social desirability bias, as participants may have underreported SLPs use due to perceived social disapproval or overreporting due to normalization within peer groups. The courses per each university were selected purposely rather than randomly; this may limit the representativeness of the sample within other institutions and generalization. The universities which were selected are coming from one urban district in Tanzania, which limits generalization to other regions and universities in Tanzania. The SLPs reported were not confirmed by the laboratory, and also to ascertain their concentration. Therefore, the presence and level of exposure to prohibited substances could not be objectively verified. The study was limited to the views of undergraduate female university students; however, the inclusion of male university students could have contributed additional insights. Furthermore, the current study focused on exploring self‐perceived skin tone as a cosmetic determinant among participants lacking standardization in dermatological research. However, this is taken as a limitation and the Fitzpatrick Skin Phototype scale will be considered in future studies.

## 6 Conclusion

This study found that nearly half (45.5%) of undergraduate female university students from the studied universities used SLPs. Use of banned products, particularly those containing hydroquinone and corticosteroids in combination, was prevalent, and the majority of users applied them daily with little or no awareness of product ingredients. Fair and white skin color and off‐campus residence were independently associated with SLPs use. These findings have clear implications for multiple stakeholders. The Tanzania Bureau of Standards and relevant regulatory authorities should intensify enforcement of existing legislation governing the sale and distribution of SLPs, including stronger monitoring of cosmetic shops and online retail channels in the Kilimanjaro region and beyond.

The studied universities should develop educational programs to raise awareness of the risks associated with prohibited SLP practices, in order to empower students to make safe choices. Future longitudinal and qualitative research is needed to better understand the behavioral determinants of SLPs' use and to evaluate the effectiveness of regulatory and educational interventions in this population.

## Author Contributions

Isaya Kweka, John Mtenga, Mariam Chumo, Jackline Njau, Lameck Pashet Sengeruan, Sia Saria, Jere Mshana and Rachel Manongi: conception and design of the work, ensuring data integrity, analysis and interpretation of the data; drafting the manuscript and revision. Daniel E. Mwakasaka: analysis and interpretation of the data, drafting the manuscript and revision. All authors have read and approved the final manuscript.

## Funding

The authors have nothing to report.

## Ethics Statement

This study was approved by the Ethics Committee at Kilimanjaro Christian Medical College (IRB No. UG27/2024). Participation was entirely voluntary, consent was obtained from each participant and being informed to withdraw from the study at any time. The data were collected for exclusive research purposes with anonymity and confidentiality being ensured.

## Conflicts of Interest

The authors declare no conflicts of interest.

## Data Availability

Data that support the findings of this study are available on reasonable request from the corresponding author.
